# Human embryonic stem cells extracellular vesicles and their effects on immortalized human retinal Müller cells

**DOI:** 10.1371/journal.pone.0194004

**Published:** 2018-03-14

**Authors:** Yingqian Peng, Edouard Baulier, Yifeng Ke, Alejandra Young, Novruz B. Ahmedli, Steven D. Schwartz, Debora B. Farber

**Affiliations:** 1 Stein Eye Institute, Department of Ophthalmology, UCLA School of Medicine, Los Angeles, CA, United States of America; 2 Molecular Biology Institute, UCLA, Los Angeles, CA, United States of America; 3 Brain Research Institute, UCLA, Los Angeles, CA, United States of America; Cedars-Sinai Medical Center, UNITED STATES

## Abstract

Extracellular vesicles (EVs) released by virtually every cell of all organisms are involved in processes of intercellular communication through the delivery of their functional mRNAs, proteins and bioactive lipids. We previously demonstrated that mouse embryonic stem cell-released EVs (mESEVs) are able to transfer their content to different target retinal cells, inducing morphological and biochemical changes in them. The main objective of this paper is to characterize EVs derived from human embryonic stem cells (hESEVs) and investigate the effects that they have on cultured retinal glial, progenitor Müller cells, which are known to give rise to retinal neurons under specific conditions. This would allow us to establish if hESEVs have a pro-regenerative potential not yet described that could be used in the future for treatment of human retinal degenerative diseases. Initially, we showed that hESEVs are heterogeneous in size, contain mRNAs and proteins involved in the induction and maintenance of stem cell pluripotency and can be internalized by cultured Müller cells. After a single exposure to hESEVs these cells display changes in their gene expression profile, and with multiple exposures they de-differentiate and trans-differentiate into retinal neuronal precursors. hESEVs were then fractionated into microvesicles (MVs) and exosomes (EXOs), which were characterized by size, specific surface proteins and biochemical/molecular components. We demonstrate that despite the similar internalization of non-fractionated hESEVs, MVs and EXOs by Müller progenitor cells, *in vitro*, only the release of MVs’ cargo into the cells’ cytoplasm induces specific changes in their levels of pluripotency mRNAs and early retinal proteins. EXOs do not produce any detectable effect. Thus, we conclude that MVs and MVs-containing hESEVs are promising agents that possibly could promote the regeneration of diseased or damaged retinas *in vivo* through inducing glial Müller cells to become replacement neurons.

## Introduction

Extracellular vesicles (EVs), membranous vesicles limited by a lipid bilayer and containing hydrophilic soluble components [1], are released by virtually every cell of multicellular organisms, including stem cells, into their extracellular space [2]. EVs are heterogeneous in size and include microvesicles (MVs, ~100–1,000 nm diameter, shed from the plasma membrane) and exosomes (EXOs, ~20–120 nm diameter, endosomal origin). EVs can transfer their content to various cell types by first interacting with cell surface receptors and then releasing their luminal components (mRNAs, microRNA and proteins) into the cytosol of the targeted cells [3]. Because of this function, EVs are considered important regulators of cell-to-cell communication.

EVs are emerging as potent genetic information transfer agents underpinning a range of biological processes and demonstrating therapeutic potential for tissue regeneration in degenerative diseases of various organs such as kidney [4, 5], heart [6], liver [7] and lung [8, 9], and stimulating ocular [10, 11] and bone [12] restoration.

Cultures of spontaneously immortalized human retinal progenitor Müller cells, the main glial population of the retina [13], when exposed to mouse ESEVs (mESEVs) experience gene expression changes associated with de-differentiation and pluripotency induction as well as activation of an early retinogenic program of differentiation [11]. Thus, ESEVs may be promising therapeutic agents capable of stimulating Müller cells to rescue the morphology and function of degenerating or damaged retinas. As first step in exploring this hypothesis, we characterized human ESEVs (hESEVs) and two of their fractionated subpopulations, MVs and EXOs. We determined the size of these vesicles, their molecular and biochemical components, the surface proteins that can distinguish them, and assessed the effects they have on human Müller cells *in vitro*. Most interesting, we observed that hESEVs and MVs differ from EXOs in their transfer of mRNAs to cultured Müller cells and that continued exposure to hESEVs or MVs induces Müller cells to trans-differentiate into retinal neuronal precursors.

## Materials and methods

### Culture of Human embryonic stem cells (hESCs)

H9 hESCs were obtained from the UCLA Broad Stem Cell Research Center Core facility, expanded under serum- and feeder-free conditions in filtered (Corning filter system, 0.22 μm polyethersulfone membrane) mTeSR1 medium (Stem Cell Technologies, Vancouver, Canada), plated on T75 or T175 culture flasks coated with Matrigel (BD Biosciences, San Jose, CA) and cultured in a humidified 37°C, 5% CO2 incubator. The culture medium was changed daily and collected every 24 hours for ESEV isolation. hESCs were passaged using dispase (Stem Cell Technologies) every 5–6 days to maintain H9 colonies at 80% confluence in order to maximize ESEV yield. hESC colonies were inspected daily by microscopy for signs of differentiation.

### hESEV isolation

Culture medium from H9 hESCs maintained at 80% confluence was spun at 3,500g for 1 hour at 4°C to pellet debris, fragmented cells and apoptotic bodies. The supernatant was then spun at 200,000g for 3.5 hours (*k-*Factor 104.8) in a Type 50.2 TI rotor (Beckman Coulter, Brea, CA) at 4°C to pellet hESEVs.

To determine whether the hESC passage number (P) influenced the quality and quantity of hESEVs released, two T175 flasks of H9 hESCs at P38, P42, P46 and P50 were cultured following the same protocol. When cells reached 80% confluence, hESEVs were collected from the same amount of medium of each hESC passage and their total RNA was isolated using the mirVana miRNA Isolation Kit (Ambion, Waltham, MA).

### Fractionation of hESEVs

hESEVs were fractionated into apoptotic bodies, MVs and EXOs by differential centrifugation. The hESC culture medium was collected after 24 hours at 80% confluence and spun at 300g to pellet debris and fragmented cells. The supernatant was centrifuged at 2,800g for 40 minutes to separate apoptotic bodies, followed by two ultracentrifugation steps (Beckman 50.2 TI rotor), the first one at 16,500g (*k-*Factor 1270.5) for 1 hour at 4°C to pellet MVs and the last one at 120,000g (*k-*Factor 131) for 2 hours at 4°C for pelleting EXOs.

### Characterization of MVs and EXOs

#### a. Size determination using TEM

After collection, MVs and EXOs were washed with 5 ml filtered (0.1 μm Acrodisc syringe filter, Pall Corporation, Port Washington, NY) buffer solution (25 mM HEPES + 0.9% NaCl in water) and centrifuged again for 1 hour at 16,500g or 2 hours at 120,000g, respectively. MVs and EXOs were then suspended in 200 μl of the same buffer and 5 μl were placed on a formvar carbon-coated 200 mesh copper grid (EMS, Hatfield, PA) for 5 minutes. The grid was placed on top of a drop of 2% uranyl acetate solution (EMS) for 3 minutes and, after blotting the excess of uranyl acetate, it was dried at room temperature before imaging either with a FEI T12 transmission electron microscope (FEI Tecnai, Hillsboro, OR) under an acceleration of 120 kV or a JEM1200-EX (JEOL, Huntington Beach, CA) under an acceleration of 80 kV. The size of more than 100 MVs or EXOs from 3 independent collections was measured with this method.

#### b. Size determination using DLS

Pellets containing MVs or EXOs were re-suspended in 50 μl filtered (Corning filter system) PBS and 20 μl of each suspension, diluted 3 times to dissociate possible hESEV aggregates, were distributed between triplicate disposable micro-cuvettes (WYATT Technology, Santa Barbara, CA). MV and EXO sizes were determined by DLS recorded for 200 seconds at 25°C using the DynaPro NanoStar detector (WYATT Technology). Ten replicate measurements were obtained for each sample.

#### c. Quantitative PCR (qPCR) measurement of pluripotency-associated mRNAs levels

Total RNA of H9 hESCs as well as of human embryonic kidney cells (HEK293, ATCC, Manassas, VA) used as negative control, was isolated using the mirVana miRNA isolation kit and treated with TURBO DNAse (Ambion) to remove DNA traces. The RNAs (150 ng /sample) were converted to cDNAs using SuperScript III First-Strand Synthesis SuperMix for reverse transcription PCR (RT-PCR, Invitrogen, Thermo Fisher Scientific) following the company’s protocol. qPCR was then carried out to determine the levels of pluripotency-associated *OCT4*, *SOX2* and *NANOG* mRNAs in each of the samples. The 25 μl total volume reactions contained 12.5 μl Maxima SYBR Green qPCR Mastermix (2X, Thermo Fisher Scientific), 1 μl of each of the corresponding 10 μM forward and reverse human-specific primers (Table 1), 9.5 μl of water and 1 μl of cDNA (20 ng). Amplification was carried out using an Eppendorf Mastercycler RealPlex2 (Eppendorf, Hauppauge, NY) as follows: 95°C for 10 minutes, 45 cycles of 95°C for 30 seconds, 60°C for 30 seconds and 72°C for 45 seconds, ending with 72°C for 10 minutes followed by 37°C for 20 minutes. In each experiment, a sample without reverse transcriptase and a sample without template were included to demonstrate specificity and lack of DNA contamination. Fold-difference in H9 hESCs’ gene expression was calculated using the 2-ΔΔCt method for each mRNA tested compared to that in HEK293 cells. Three biological replicates were run in parallel for each sample.

**Table 1:** Human-specific primer pairs used to amplify the indicated mRNAs.

mRNA	Forward Primer	Reverse Primer
***OCT4***	CAGGGTTTTTGGGATTAAGTTCT	TGTGTCCCAGGCTTCTTTATTTA
***SOX2***	AAATGACAGCTGCAAAAGAGAAC	GATCTATACAAGGTCCATTCCCC
***NANOG***	CTGCCGTCTCTGGCTATAGATAA	CACAACCAACAAATTAGGGGAA
***GAPDH***	AAGCTCATTTCCTGGTATGACAA	TGGTTGAGCACAGGGTACTTTAT
***PAX6***	CGGAAGTGAACCTGATATGTCTC	AACAGCCATTTTTCTTTCTTTCC
***RAX***	AAACTGTCAGAGGAGGAACAGC	GACCTCTGGTAGGTTGACCTTG
***KLF4***	GGTACCAAACAAGGAAGCCA	TGCTTAAGGCATACTTGGGAA
***PI4K2A***	TAGTGCCATTGACCGAGTGA	CGGATGATGTAATCCAGCAC
***CECR7***	GAGGGTGCATCCTGAGAGAG	GATCGCCCACTGAAGTGAAT
***SLC7A3***	GCTATCTACTTCGGCTATGGGAT	CACATCCTTCACATCGTACAGAA

All primers were designed using Primer3 (http://simgene.com/Primer3) and synthesized by IDT, Coralville, IA.

#### d. Microarray hybridization and data analysis

Total RNA (200 ng) was profiled for each of three biological samples of hESEVs, MVs and EXOs at the UCLA Clinical Microarray Core facility using the Affymetrix® HG-U133 Plus 2.0 Arrays (Affymetrix, Santa Clara, CA) following the manufacturer’s instructions. Total RNA was first reverse-transcribed into cDNA and next, second strand cDNA was synthesized. Following *in vitro* transcription of the cDNA, 12.5 μg of amplified and purified RNA were labeled with biotin and fragmented prior to hybridization to the arrays, which was performed at 45°C for 16 hours in an Affymetrix hybridization oven. The arrays were then washed and stained with Streptavidin Phycoerythrin in the Affymetrix Fluidics station (model 450) and scanned with the Affymetrix 7G scanner. The Affymetrix GeneChip Command Console 4.0 (AGCC) was used for controlling the system and for acquisition of array images. Data was processed with Affymetrix MAS5 software, which includes scaling all probes to a target intensity (*TGT*) of *500*. Data was further analyzed with Partek Genomics Suite software. We used the RMA algorithm for data normalization. Thresholds for selecting genes expressed at much higher levels in MVs than EXOs or vice-versa were set at ≥ 2.0-fold difference and p<0.05. Global functional analyses, network analyses and canonical pathway analyses were performed using the Ingenuity Pathway Analysis (Ingenuity Systems, www.ingenuity.com). The accession number for the microarray data deposited in GEO is: GSE 102176.

#### e. Validation of microarrays results

RT-qPCR of total RNA from MVs, EXOs and hESEVs samples was carried out as described above (c) using specific primer pairs for the selected mRNAs to be evaluated (Table 1). Since microarray data showed *GAPDH* mRNA had a disparity in expression levels in MVs and EXOs, we could not use *GAPDH* mRNA as the endogenous control normalizer in comparative quantification experiments. Instead, we relied on adding equivalent amounts of RNA from MVs, EXOs and hESEVs samples in each experiment and used the non-fractionated hESEVs (which contain both MVs and EXOs) as normalizer. Three biological replicates were run in parallel for each sample.

#### f. Western blot analysis

24 hours prior to the isolation of hESEVs, MVs and EXOs for Western blots, the mTeSR1 medium used for culturing H9 hESCs was replaced by TeSR-E8, a low-protein formulation medium (Stem Cells Technologies). For these experiments, the culture medium of 4 T175 flasks containing 80% confluent H9 cells was collected every 24 hours for 2 consecutive days. All vesicles and the H9 cells from which they originated were digested in RIPA buffer (20 mM Tris-HCL, 150 mM NaCl, 1% NP-40, 1% Na^+^ deoxycholate) with protease inhibitors cocktail (cOmplete mini, Roche, Basel, Switzerland) for 2 hours at 4°C. Protein concentration was assessed using the micro BCA kit (Thermo Fisher Scientific).

When Western blots were used to detect Müller cell proteins after treatment with hESEVs, protein concentration was estimated using the Bradford assay. For each experiment, 25–30 μg protein were denatured at 65°C for 10 minutes in reducing conditions and resolved on a 4–12% Bis-Tris SDS gel (Thermo Fisher Scientific) with MOPS buffer (Bioland Scientific, Paramount, CA). β-ACTIN or GAPDH was used as loading control. Proteins were then transferred to a PVDF membrane (Bio-Rad, Irvine, CA) and blocked 1 hour at room temperature with Odyssey PBS blocking buffer (Li-Cor Biosciences, Lincoln, NE). Membranes were sequentially incubated with antibodies against CD9, CD63, CD81, CD90, GAPDH, OCT4, SOX2 and β-ACTIN (Table 2), followed by the corresponding secondary antibodies (donkey anti-mouse, donkey anti-rabbit or donkey anti-mouse IR-Dye 670rd or 800cw, Li-Cor). Blotted membranes were imaged using the Odyssey CLX imaging system (Li-Cor).

**Table 2:** Antibodies list.

Target	Host	Clone	Brand/catalogue #	Application	Dilution
**CD9**	Rb	EPR2949	Abcam, ab92726	WB	1:1000
**CD63**	Rb	Polyclonal	Abcam, ab118307	WB	1:500
**CD81**	Ms	1.3.3.22	Thermo Fisher, MA5-13548	WB	1:100
**CD90**	Rb	EPR3132	Abcam, ab92574	WB	1:1000
**GAPDH**	Rb	FL-335	Santa Cruz, SC-25778	WB	1:2000
**OCT4**	Gt	Polyclonal	Abcam, ab27985	WB ICC	1:1000 1:500
**SOX2**	Rb	Polyclonal	Abcam, ab97959	WB ICC	1:200 (H9 cells & ESEVs) 1:1000 (Müller cells) 1 :1000
**β-ACTIN**	Ms	AC-15	Abcam, ab6276	WB	1:5000
**NANOG**	Rb	D73G4	Cell Signaling Technology, 4903	WB ICC	1:2000 1:400
**PAX6**	Ms	AD2.38	Abcam, ab78545	ICC	1:500
**TRA1-81**	Ms	tra-1-81	Thermo Fisher, MA1-024X	ICC	1:200
**VIMENTIN**	Ms	V9	Bio-Rad, MCA862	ICC	1:500

Primary antibodies for Western blots (WB) were diluted in PBS/Li-Cor blocking buffer containing 0.1% Tween 20. Primary antibodies for immunocytochemistry (ICC) were diluted with PBST containing 10% of the serum corresponding to the secondary antibody. Gt: Goat; Ms: mouse; Rb: Rabbit. For the staining of CD81, the samples were denatured in non reducing conditions prior to electrophoresis. The membrane was subsequently blocked in TBS/Li-Cor blocking buffer. The primary antibody against CD81 was diluted in TBS/Li-Cor blocking buffer containing 0.1% Tween 20.

A list of antibodies used for Western blots of hESEVs, MVs and EXOs, as well as for Müller cells that had been treated with these nanovesicles, is in Table 2.

### Culture of retinal Müller and embryonic kidney cells

The spontaneously immortalized human Moorfield/Institute of Ophthalmology-Müller 1 cell line (MIO-M1), initially derived from postmortem human retina, was established, characterized [14] and demonstrated to express markers of neural progenitors [15]. MIO-M1 Müller and HEK293 cells were cultured in 1X DMEM/F-12 (1:1) medium containing 2.5 mM L-glutamine, 15 mM HEPES (Thermo Scientific HyClone, Loughborough, UK) and 10% vol/vol fetal bovine serum (filtered, heat inactivated; Gemini Bioproducts, Sacramento, CA) in T175 flasks. Cells were kept in a humidified 5% CO2 incubator at 37°C until collection for RT-qPCR experiments.

### Cultured Müller cells uptake of MVs and EXOs detected by imaging flow cytometry

MVs and EXOs fractionated from the hESC medium of 1 T175 flask as described above were immediately re-suspended in tubes containing 1 ml of lipophilic carbocyanine DiO solution (5 μM, Thermo Fisher Scientific) and labeled for 30 minutes at room temperature. After adding 10 ml PBS to each tube, MVs or EXOs were collected after ultracentrifugation at 16,500g for 1 hour at 4°C and at 120,000g for 2 hours at 4°C, respectively. Müller cells were cultured in 6-well plates (100,000 cells/well) until they reached 50% confluence; then, the labeled MVs or EXOs were added (control Müller cells received Müller culture medium only). Cells were incubated for 8, 12, 24 and 36 hours. At the appropriate time, treated and control Müller cells were dissociated with 0.25% trypsin and re-suspended in 50 μl PBS for imaging on an ImageStream X MkII fIow-cytometer (Amnis Corporation, Seattle, WA). Channel 04 was used for collecting intensity-adjusted bright-field images, channel 02 for fluorescence images and channel 06 for side-scattered light. 488 nm and 785 nm lasers were activated for fluorescence and side-scatter, respectively. The laser power was 200 mV in channel 02 and 2 mV in channel 06. INSPIRE software was used with settings at 40X magnification and flow rate at low speed/high sensitivity. To determine the uptake of MVs or EXOs into single Müller cells, a scatter plot was created with cell area on the X- axis against aspect ratio (this is the Minor Axis divided by the Major Axis of cells, which describes how round or oblong the cells are) in the Y- axis. From this scatter plot, a small area and a high aspect ratio was selected to obtain a single cell. The IDEAS Analysis Software (Version 4.0) was used to analyze the data. First, a mask (it defines a specific area of an image to use for calculations) was created to localize whole cells. For this, we utilized the AdaptiveErode (a mask that identifies pixels that will form a circle that touch the input boundary of the object) to circle the cell. After that, spot count was added to the AdaptiveErode mask to count spots inside of the Müller cells and the intensity of the spots was measured.

### Treatment of Müller cells with H9 hESEVs, MVs or EXOs

Müller cells were plated on 6-well cell culture plates at 50,000 cells per well and allowed to reach 50–60% confluence prior to initiating hESEV treatments. For these, non-fractionated hESEVs pelleted by ultracentrifugation of media from 7 T75 flasks of H9 hESCs (passages 38 to 42), were each re-suspended in 3 ml of Müller cell medium and then added to 3 wells (treated cells). The other 3 wells received an equal volume of Müller cell medium (control cells). Cells were cultured in a humidified 5% CO2 incubator at 37°C for 8 hours, washed with ample PBS and their total RNA isolated and converted to cDNA for qPCR measurements. The same experiment was carried out incubating Müller cells with hESEVs for 24, 36 and 48 hours. The 24-hour incubation time was chosen to perform Western blot analyses of the treated and control Müller cells. In addition, similar experiments were performed replacing the hESEVs with MVs or EXOs obtained from media of 9 T175 flasks.

To determine the effect of repeated treatments of Müller cells, cultures were exposed to freshly isolated hESEVs every 48 hours using the same protocol up to 13 times. Control cells were subjected only to medium changes. Both treated and control cultures were passaged and split at a 1:3 ratio at the end of every second hESEV treatment. hESEVs, MVs and EXOs-exposed and control Müller cells were examined by microscopy after each treatment.

#### qPCR of Müller cell mRNAs

Human-specific primer pairs for *OCT4*, *SOX2*, *NANOG*, *PAX6* and *RAX* mRNAs (Table 1) and the SYBR Green I Master reaction mix (Thermo Fisher Scientific) were used for qPCR analysis of gene expression changes in Müller cells post-hESEVs, MVs or EXOs treatment. Amplification was detected with the LightCycler 480 System in treated and control Müller cells. All results were normalized to the human *GAPDH* mRNA. The relative change in gene expression was determined using the 2-ΔΔCt method of comparative quantification.

#### Immunocytochemistry

To investigate stem cell and retina cell-specific markers expressed in control and hESEVs-, MVs- or EXOs-treated Müller cells as well as to assess the stability of hESCs at different passages we used immunocytochemical methods and antibodies listed in Table 1. Control and 12-times treated with hESEVs, MVs or EXOs Müller cells were seeded on poly-D-lysine-coated (10 μg/ml, Sigma-Aldrich, St. Louis, MO) glass coverslips placed in 24-well culture plates. Cells were allowed to attach, and then the treated cells were exposed one more time to hESEVs, MVs or EXOs (13^th^ treatment). 24 hours later, hESEV-treated and control cells were rinsed with 0.01 M PBS and fixed for 10 minutes in ice-cold methanol, rinsed again in 0.01 M PBS and, depending on the secondary antibody used, blocked with 10% donkey or goat serum in PBST (0.01 M PBS + 0.1% Tween 20) for 1 hour at room temperature. Cells were incubated with primary antibodies (Table 2) overnight, at 4°C, followed by 1 to 2 hours incubation at room temperature with the appropriate secondary antibodies diluted 1:500 in PBST containing 10% serum and conjugated to Alexa Fluor 488, Alexa Fluor 568, or Alexa Fluor 594 (Molecular Probes/Thermo Fisher, Eugene, OR). Cells were then washed extensively with PBST and incubated with DAPI (Invitrogen). Coverslips were mounted on slides using Fluoromount-G (Southern Biotech, Birmingham, AL), allowed to dry, and images were obtained with an Olympus FluoView FV1000 confocal laser-scanning microscope using Olympus FluoView software for capture and processing (Olympus America, Center Valley, PA).

To assess the stability of H9 ESCs during their culture on T175 flasks under feeder-free and serum-free conditions, cells (passages P41 and P50) were cultured on Matrigel-coated coverslips in mTeSR1 medium for 2–3 days after passaging, rinsed in 0.01 M PBS, fixed for 15 minutes in 4% PFA at room temperature, and rinsed twice in 0.01 M PBS. The following steps of blocking, staining, mounting and imaging were conducted applying the same methods used for human Müller cells.

### Statistical analyses

When two groups were compared, unpaired Student’s t-test was used to assess significance of the difference between those groups. For the S1D Fig, the statistical analysis was performed using SAS, version 9.3. The RNA level in the 4 groups was compared using a repeated measures ANOVA model, and a p = 0.0008 was determined for the overall difference across all groups. Since this difference was statistically significant, a pairwise comparison between any two groups within the ANOVA model was also carried out.

## Results

### Cultured H9 hESCs maintain high levels of pluripotency mRNAs and proteins after many passages while hESEVs released from older H9 hESCs have lower levels of total RNA

Gene expression analysis performed on cultured human H9 ESCs from different passages (P38, P42, P46 and P50) showed that these cells have similar levels of pluripotency mRNAs *OCT4* (~2800-fold), *NANOG* (~800-fold) and *SOX2* (~14-fold) relative to the minimal detectable amounts present in negative control HEK293 cells (S1A Fig). Furthermore, incubation of fixed and blocked P41 and P50 H9 hESCs with antibodies against TRA 1–81, SOX2, OCT4 and NANOG (Table 2) showed high expression of these proteins in all cells (S1B and S1C Fig, respectively). This indicates that the H9 hESCs’ pluripotent state is preserved during the culture process. Interestingly, however, the S1D Fig shows that total RNA levels in hESEVs released from H9 hESCs at different passages remain stable between P38 and P42 (3000 ± 112 ng and 3104 ± 14 ng, respectively), but decrease at P46 (2064 ± 20 ng) and are quite lower at P50 (832 ± 40 ng). No RNA was found in the mTeSR1 medium in which the cells were cultured. Therefore, we only used passages earlier than P44 to conduct all experiments using hESEVs.

### Characterization of hESEVs

#### hESEVs contain pluripotency mRNAs

**Fig 1:**
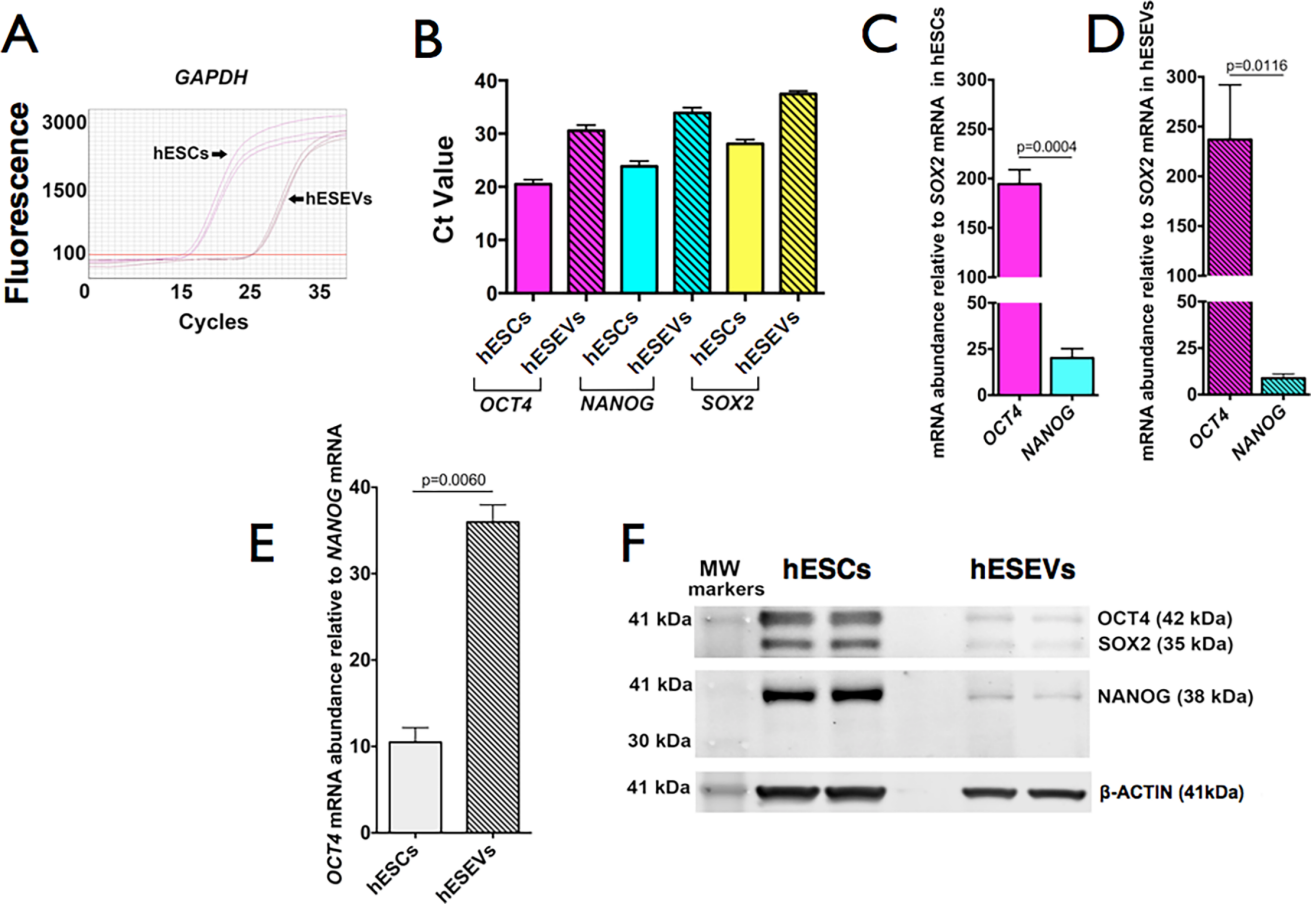
Expression levels of pluripotency mRNAs and proteins in hESCs and hESEVs. (A) qPCR amplification curves for *GAPDH* mRNA reveal much less expression in hESEVs compared with hESCs. (B) mRNAs for *OCT4*, *NANOG* and *SOX2* show higher Ct values for hESEVs than for hESCS. (C) Relative abundance of *OCT4* and *NANOG* mRNAs to *SOX2* mRNA in hESCs and (D) in their released hESEVs. (E) The OCT4/NANOG ratio is 3.5-fold higher in hESEVs than in hESCs. (B), (C), (D) and (E) bars represent the mean ± SEM from data of 3 different quantifications. p-values were calculated using the Student’s t-test. (F) Representative Western blot (n = 3) using similar amounts of protein from hESCs or hESEVs per lane and primary antibodies against OCT4, SOX2, NANOG and β-ACTIN proteins.

hESEVs derived from H9 hESCs contain *OCT4*, *SOX2* and *NANOG* mRNAs. To compare the levels of these mRNAs in hESEVs and hESCs, a similarly expressed housekeeping gene’s mRNA is needed as a normalizer. However, we found that the qPCR curves for *GAPDH* (Fig 1A) and β*-ACTIN* (not shown) mRNAs, obtained using the same amount of hESC and hESEV total RNA, had a higher Ct value (lower mRNA level) for hESEVs than for hESCs. Thus, these mRNAs could not be used for the comparative quantification of hESEVs’ and hESCs’ mRNAs. Instead, we determined the relative abundance of the 3 pluripotency mRNAs with RT-qPCR measurements using the same amount of hESC and hESEV total RNA and human-specific primer pairs for each mRNA (Table 1). *OCT4*, *NANOG* and *SOX2* mRNAs are present at higher levels in hESCs than in hESEVs (Fig 1B), with *SOX2* being the lowest of the three mRNAs in both samples. We then compared *OCT4/SOX2* and *NANOG/SOX2* mRNA ratios in hESCs and hESEVs. Our results show that *OCT4* mRNA level is 194 ± 15 –fold higher than that of *SOX2* mRNA in hESCs (Fig 1C) and 237 ± 55-fold higher than that of *SOX2* mRNA in hESEVs (Fig 1D), whereas *NANOG* mRNA levels are 20 ± 5 –fold higher in hESCs and 7 ± 1 –fold higher in hESEVs than the respective levels of *SOX2* mRNA (Fig 1C and 1D). The lack of statistically significant difference between the *OCT4* to *SOX2* mRNA relative abundance in hESCs and hESEVs suggests that *OCT4* and *SOX2* mRNAs are incorporated into the hESEVs in the same proportion as they are found in the hESCs. On the other hand, the *NANOG/SOX2* ratio is lower in hESEVs than in hESCs (Fig 1C and 1D), possibly indicating that *NANOG* mRNA is less stable in the hESEVs or selectively excluded from them as they are released from the hESCs. Thus, the *OCT4* mRNA level in hESCs is 10.49 ± 2.9– and in hESEVs 35.97 ± 1.99–fold higher than that of *NANOG* mRNA (Fig1E).

#### hESEVs carry pluripotency transcription factors OCT4, SOX2 and NANOG

Western blots show that the amount of SOX2, OCT4 and NANOG proteins is less in hESEVs than in hESCs (Fig 1F). The content of β-ACTIN is also different in hESEVs and hESCs; thus, it cannot be used as a normalizer in experiments that compare protein components of hESEVs and hESCs.

### Fractionation of hESEVs

hESEVs present in the conditioned medium of hESCs were fractionated by differential ultracentrifugation into MVs and EXOs, which were characterized by transmission electron microscopy (TEM), dynamic light scattering (DLS), Western blots, total RNA electrophoresis, microarrays of cDNAs and presence of surface marker proteins.

#### MV and EXO populations are heterogeneous in size

**Fig 2:**
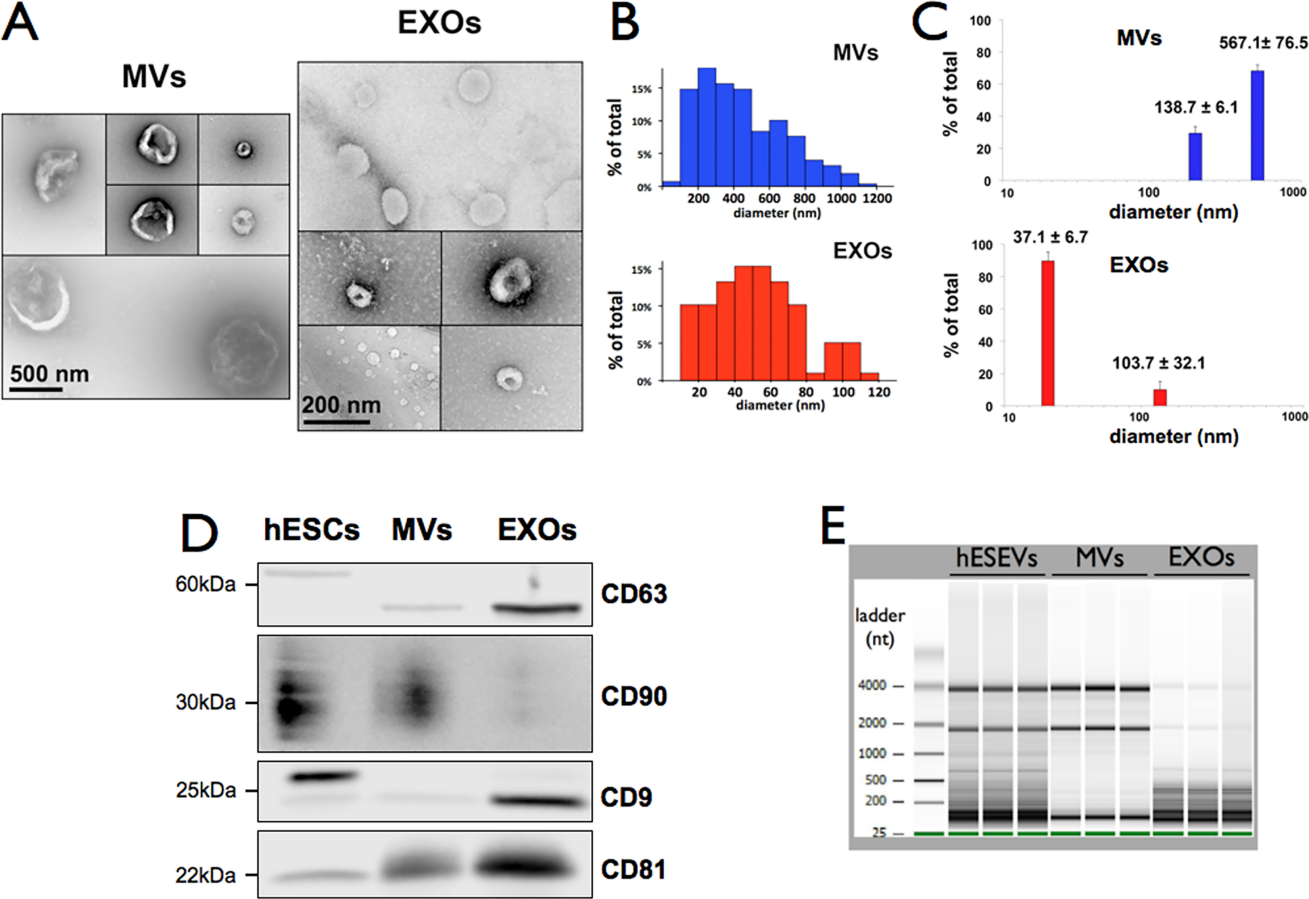
Size, markers and total RNA profiles of MVs and EXOs. (A) TEM images of H9 hESC-derived MVs and EXOs after negative staining with uranyl acetate. Scale bars: MVs, 500 nm and EXOs, 200 nm. (B) Bar graphs show vesicles’ size distribution (diameter) in the TEM images of MVs and EXOs from 3 independent hESEV fractionations. (C) Diameters of the two main MV and EXO populations from 3 independent hESEV fractionations recorded by light scattering. Numbers above bars are the mean ± SEM. (D) Representative Western blot of CD9, CD63, CD81 and CD90 in H9 hESCs and in the MVs and EXOs derived from them. (E) Electrophoresis of total RNA from hESEVs, MVs and EXOs.

TEM showed that the diameter of MVs ranges from 96 nm to 1.1 μm. EXOs are much smaller, with diameters between 16 to 112 nm (Fig 2A). Both nanoparticle populations are distributed into two major groups (Fig 2B), MVs: ~ 200 and 600 nm and EXOs ~ 40 and 100 nm diameters. DLS confirmed the TEM results, showing two populations for each type of nanoparticles (Fig 2C): MVs, 138.7 ± 6.1 nm and 567.1 ± 76.5 nm and EXOs, 37.1 ± 6.7 nm and 103.7 ± 32.1 nm.

#### MVs and EXOs show different expression of CD9, CD63, CD81 and CD90 surface markers

The tetraspanins CD9 and CD63 are exosomal markers that have different isoforms with distinct molecular weights: 22 to 27 kDa for CD9 [16, 17] and 26, ~53 and ~60 kDa for CD63 [18, 19]. CD81 is a tetraspanin detected in both MVs and EXOs, with higher expression in the latter [20]. CD90, also called THY-1, is a heavily glycosylated phosphatidylinositol (GPI)-anchored protein (molecular weight 25-35kDa [21]). Fig 2D shows that hESCs, used as positive control for MVs and EXOs, express CD9 (~27kDa isoform), CD63 (~61 kDa isoform), CD81 (~22 kDa) and CD90 (~29–35 kDa isoforms). MVs released by these cells contain CD81, but at lower level than EXOs, and are significantly enriched in CD90 when compared to EXOs. On the contrary, EXOs originating from the same hESCs are enriched in CD9 (~24 kDa isoform), CD63 (~56kDa isoform) and CD81 compared to MVs.

#### RNA expression patterns in MVs and EXOs are different

Electrophoresis of total RNA from H9 hESCs-derived MVs and EXOs showed that MVs have much longer RNAs than EXOs, the majority ~ 2000nt to 4000nt long, while EXOs are rich only in smaller RNAs, less than 500nt long (Fig 2E). In addition, both MVs and EXOs have a population of RNAs about 50nt long, most probably consisting of miRNAs. Non-fractionated hESEVs carry all RNAs found in MVs and EXOs.

#### Microarray analyses of genes from H9 hESCs-derived MVs and EXOs show that many of them are differentially expressed

**Fig 3:**
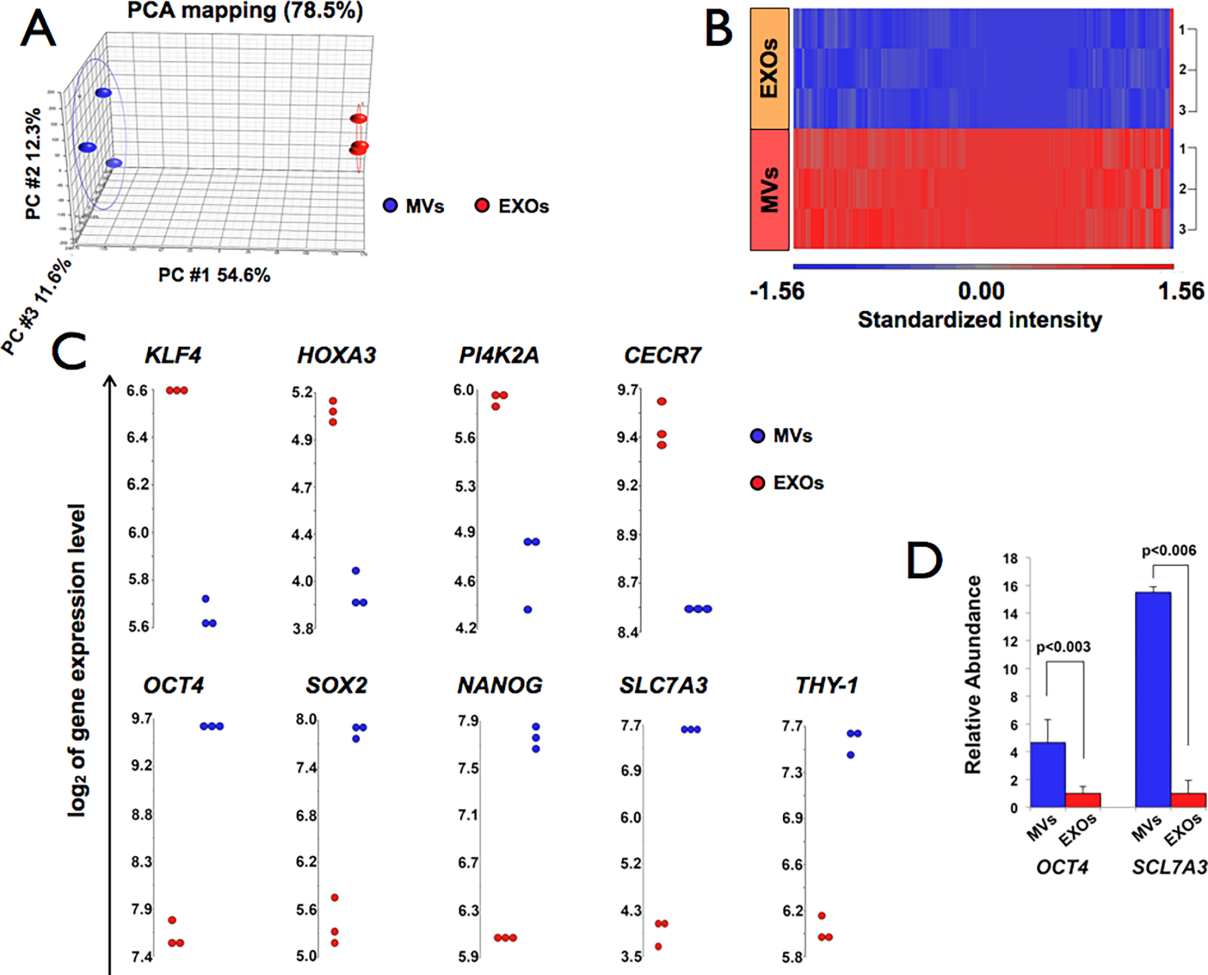
Microarray analyses of genes from H9 hESCs-derived MVs and EXOs. **(**A) Principal Component Analysis (PCA) shows differential expression levels of transcripts in MVs (blue spheres) *vs* EXOs (red spheres); (n = 3). Each dot represents a sample. PC: Principal Component. (B) Heat map representation of hierarchical clustering of 6 samples (3 of MVs and 3 of EXOs). Rows represent samples tested and columns, single genes. Red indicates higher and blue lower level of expression relative to the sample mean. (C) Dot plots of some significantly, differentially expressed genes in MVs and EXOs. Each dot plot shows the distribution of intensities for a single gene across all samples; each dot represents a sample. All analyses were done using the Partek Genomics Suite software. (D) qPCR analysis of two of the microarray-identified genes (*OCT4* and *SLC7A3)*, which were expressed at higher levels in MVs than in EXOs. Blue bars represent the mean of the relative abundance in MVs to EXOs and error bars are the SEM. p-values are shown in the figure; n = 3 in each group.

Affimetrix HG-U133 Plus 2.0 Arrays were hybridized to RNA from MVs and EXOs released from three hESCs cultures. A Principal Component Analysis (PCA) plot based on the expression levels of 54,675 probesets gave a 3D visualization of the relationship between the samples and captured 78.5% of the overall variability observed in the experiment. This analysis also showed that global gene expression patterns in biological replicates of MVs and EXOs cluster separately, indicating a clear difference in the molecular composition of the two groups of nanoparticles (Fig 3A). A hierarchical clustering heat map (Fig 3B) demonstrates distinctive patterns for MVs and EXOs and 3,274 differentially expressed genes (p-value lower than 0.05 and a minimum of 2-fold difference in expression). The majority of them (3,243) are present at higher levels in MVs than in EXOs; only 31 genes are at higher levels in EXOs than in MVs (right side of Fig 3B). Given our interest in eye diseases, we selected from the differentially expressed genes those that are known to be present in ocular tissues, and used dot plots to visualize their mRNA levels in MVs and EXOs. The upper and lower panels of Fig 3C show as examples dot plots of *KLF4*, *HOXA3*, *PI4K2A* and *CECR7* mRNAs, which are 2.0-, 2.2-, 2.6- and 2.0-fold higher in EXOs than in MVs, and of *OCT4*, *SOX2*, *NANOG*, *SLC7A3* and *THY-1* mRNAs, which are 4.2-, 5.5-, 3.3-, 13.1-, and 2.8-fold higher in MVs than in EXOs. Most of the triplicates are very close to each other, demonstrating a low experimental variability and making the difference of expression level in these genes significant.

#### Validation of microarray results

RT-qPCR of mRNAs for which we had obtained dot blots (see Table 1 for primer pairs used) was carried out to validate the results obtained with the microarray experiments. Fig 3D shows that *OCT4* and *SLC7A3* mRNAs are ~4.4- and 15.5–fold higher in MVs than EXOs, respectively, similar to the levels determined by microarrays.

#### Gene ontology analysis

Many of the genes differentially expressed in MVs versus EXOs were grouped according to the function of their expressed proteins, including RNA post-transcriptional modification, cellular growth and proliferation, protein synthesis, cell death and survival, RNA trafficking, embryonic development, and cell to cell signaling. In addition, genes were grouped according to disease type in which they may be involved, i.e., developmental disorders and neurological or ophthalmic diseases (S2 Fig). Furthermore, many of these genes were linked to canonical signaling pathways like EIF2 [22], mTOR [23] and VEGF [24] signaling (S3 Fig), which have a much lower p-value in MVs than in EXOs. In contrast, HIPPO signaling [25] has lower p-value in EXOs than in MVs (S3 Fig).

### Treatment of Müller cells with hESEVs, MVs or EXOs

Experiments were carried out using human unfractionated ESEVs and MVs or EXOs to determine the effects they had on cultured human Müller cells, since we had shown that exposure to mouse ESEVs caused up or down regulation of specific genes in these progenitor cells [11].

#### Müller cells internalize MVs and EXOs

**Fig 4:**
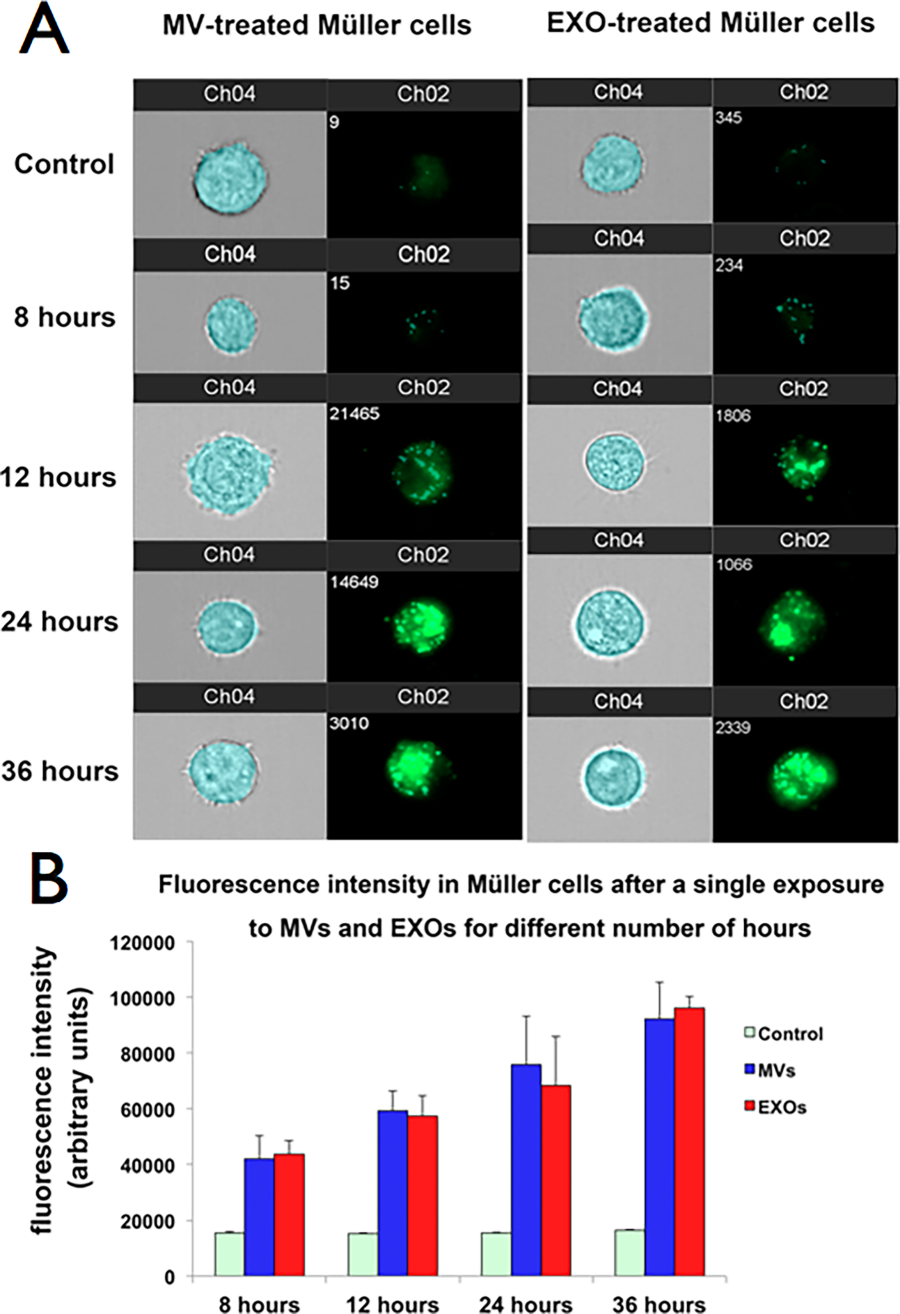
MVs and EXOs uptake by cultured human Müller cells is similar and time dependent. (A) Imaging flow-cytometry of single human Müller cells exposed to MVs or EXOs for 8, 12, 24, and 36 hours. Channel 02 shows the spot fluorescence intensity of the vesicles internalized by the cells and channel 04 the bright field images of the analyzed cells. A representative control cell cultured without exposure to MVs or EXOs is shown at the top of both panels; control cells were also analyzed at each exposure time point. (B) Quantification of the spot fluorescence intensity in human Müller cells after exposure for 8, 12, 24, and 36 hours to DiO-labeled MVs or EXOs. Bars are the mean of fluorescence intensity (arbitrary units) ± SEM. (n = 3).

Cultured Müller cells were exposed to DiO-labeled MVs or EXOs for 8, 12, 24 and 36 hours and imaging flow cytometry was used to demonstrate the nanoparticle uptake (Fig 4A). Untreated cells (control) had minimal fluorescence (15,392 ± 307 arbitrary units) measured by the intensity-spot feature of the IDEAS software, whereas the fluorescence inside cells exposed to MVs or EXOs gradually increased as a function of time of incubation. After 8 hours, only a few bright green dots were observable (fluorescence intensity of 42,048 ± 8,210 in MV-treated cells and 43,809 ± 4,709 in EXO-treated cells). Thereafter, the fluorescence intensity increased linearly reaching at 36 hours 92,190 ± 13,127 or 96,037 ± 4,199 in MVs- or EXOs-exposed Müller cells, respectively (Fig 4B). Overall, this experiment showed that Müller cells similarly internalize MVs and EXOs in a time-dependent process. They also internalize hESEVs in the same way (data not shown).

#### hESEVs induce changes in human Müller cells’ mRNAs

**Fig 5:**
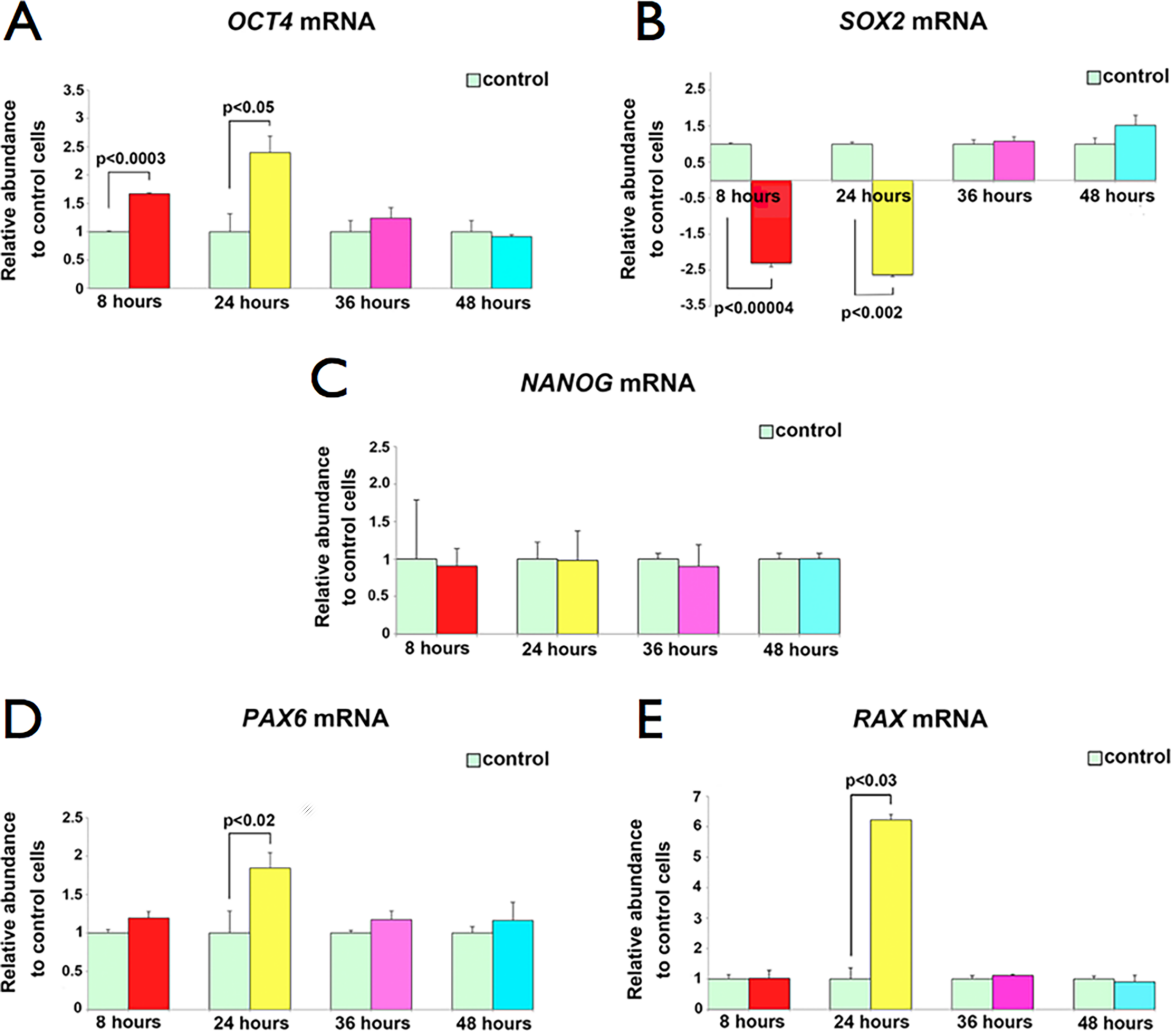
A single exposure to hESEVs changes Müller cells’ pluripotency and early retinal mRNA levels. Bar plots show the fold-change in expression for *OCT4* (A), *SOX2* (B) and *NANOG* (C) and early retinal *PAX6* (D) and *RAX* (E) mRNAs in Müller cells exposed for 8, 24, 36 and 48 hours to hESEVs, compared to expression in control Müller cells that received culture medium changes only (light green bars). All RT-qPCR results were normalized to the corresponding levels of human *GAPDH* mRNA in treated and control Müller cells. Data represent the mean ± SEM, (n = 3). p-values were calculated using the Student’s t-test.

A single exposure of cultured glial progenitor Müller cells to hESEVs for 8, 24, 36 and 48 hours caused transient changes in their levels of *OCT4*, *SOX2*, *PAX6* and *RAX* mRNAs, with maximal effect at 24 hours post-treatment. RT-qPCR measurements showed that compared to untreated Müller cells, *OCT4* mRNA is already elevated 1.7-fold after 8 hours, peaks at 24 hours with a 2.4-fold change and decreases to control levels by 36 hours (Fig 5A). In contrast, *SOX2* mRNA is reduced 2.3-fold after 8 hours of hESEV exposure, reaches its lowest level, 2.6-fold lower than control, at 24 hours and by 36 hours is back to control levels (Fig 5B). Conversely, hESEV treatment of Müller cells had no effect on *NANOG* mRNA levels. (Fig 5C). The mRNAs of early retinal genes *PAX6* and *RAX*, which encode proteins known to be expressed throughout retinogenesis by multipotent retinal progenitor cells [26, 27] are elevated in Müller cells by 1.9- and 6.2-fold, respectively, after 24 hours of hESEV exposure and decrease to control levels by 36 hours (Fig 5D and 5E).

#### A single exposure to hESEVs changes the levels of OCT4 and SOX2 proteins in cultured Müller cells

**Fig 6:**
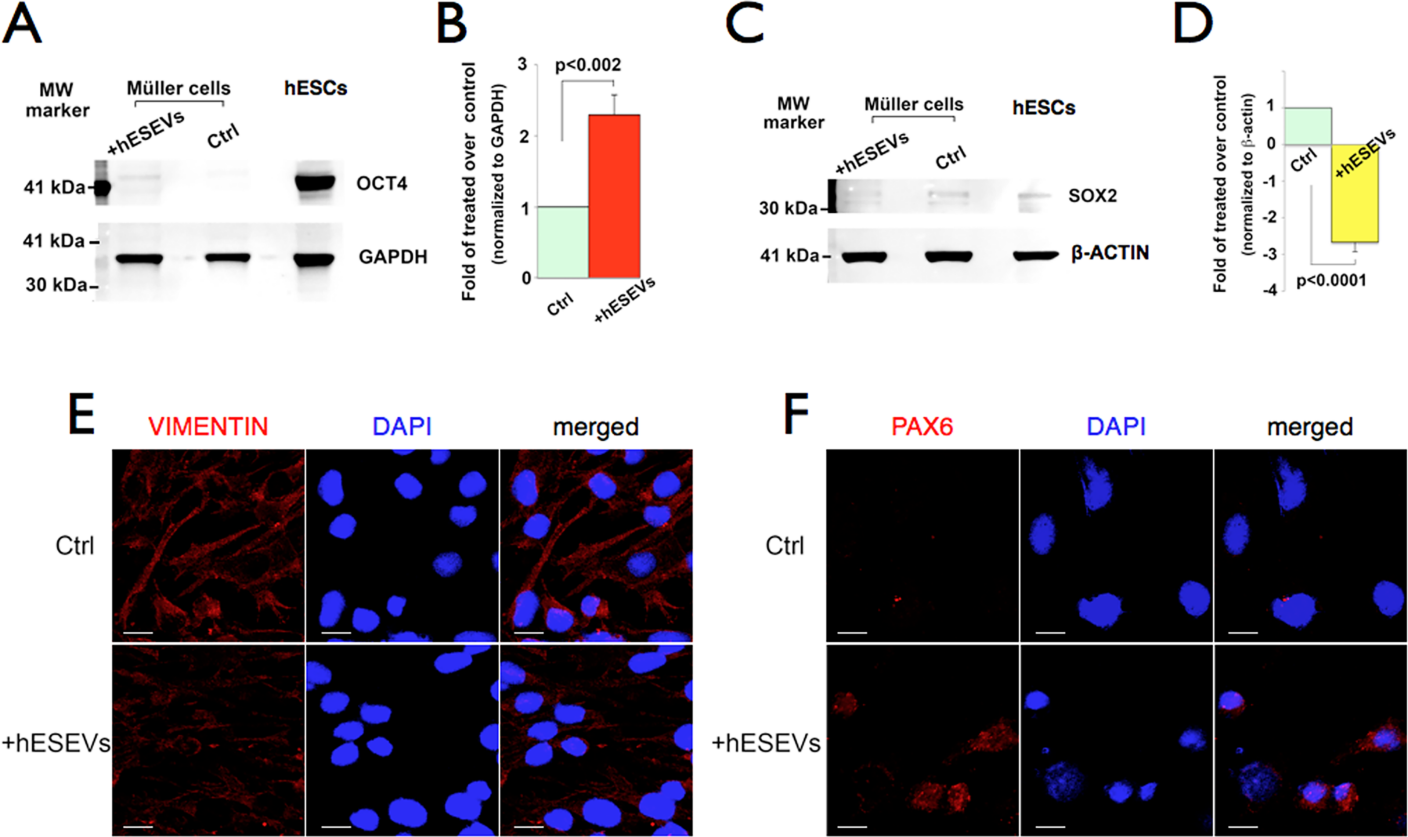
OCT4 and SOX2 protein levels in Müller cells change after 24 hours exposure to hESEVs. Extracts from control and hESEV-treated Müller cells were subjected to Western blot analyses. (A) Representative image of a blot incubated with an OCT4 antibody. The ~42kDa OCT4 protein is mainly detected in hESEV-exposed Müller cells. hESCs: positive control; GAPDH: protein loading control. (B) Quantification of OCT4 bands intensity in control and 24 hours-hESEV-exposed Müller cells, each normalized to the corresponding GAPDH protein levels. Bars show the fold-change in OCT4 protein calculated as the ratio of normalized OCT4 levels in treated and control Müller cells. (C) Representative image of a blot using a SOX2 antibody. A reduced level of the ~35kDa SOX2 protein is detected in hESEV-treated compared to untreated Müller cells. H9 cells: positive control; β-ACTIN: loading control. (D) Same as (B) but SOX2 was normalized with β-ACTIN. In (B) and (D), n = 3. Bars represent the mean ± SEM. p-values were calculated using the Student’s t-test. Ctrl: Control, untreated cells. MW: Molecular Weight. (E and F) Immunocytochemistry of Müller cells after 13 repeated exposures to hESEVs. Confocal microscopy images of control and hESEV-treated Müller cells immunolabeled with antibodies against VIMENTIN (E, red) or PAX6 (F, red). Cell nuclei were labeled with 496-diamidino-2-phenyl-indole (DAPI, blue). Magnification: 40X, scale bar 20 μm for all panels.

Western blot analysis corroborated the RT-qPCR results described above. H9 cells were used as positive controls for the expression of OCT4 and SOX2. After 24 hours of exposure to hESEVs, Müller cells expressed higher level of OCT4 than untreated cells (Fig 6A). Quantification of the blot bands using the Odyssey imaging system showed OCT4 increased ~ 2.3 fold in treated cells (Fig 6B). In contrast, SOX2 protein levels in control Müller cells were considerably decreased after 24 hours of hESEV treatment (Fig 6C). This was confirmed by quantification of the Western blot bands, which showed an ~ 2.7-fold lower expression of SOX2 in hESEV-treated Müller cells than in control (Fig 6D).

#### Repeated treatments with hESEVs induce changes in Müller cells

Immunocytochemistry of human Müller cells subjected to 13 consecutive exposures to freshly isolated hESEVs showed that the treatment induced a decrease in expression of the specific Müller cell-marker VIMENTIN (Fig 6E) and an increase in expression of the early retinal protein PAX6 (Fig 6F), compared to unexposed Müller cells.

#### Only exposure of cultured Müller cells to MVs changes their levels of *OCT4*, *SOX2* and *PAX6* mRNAs. EXOs have no effect

**Fig 7:**
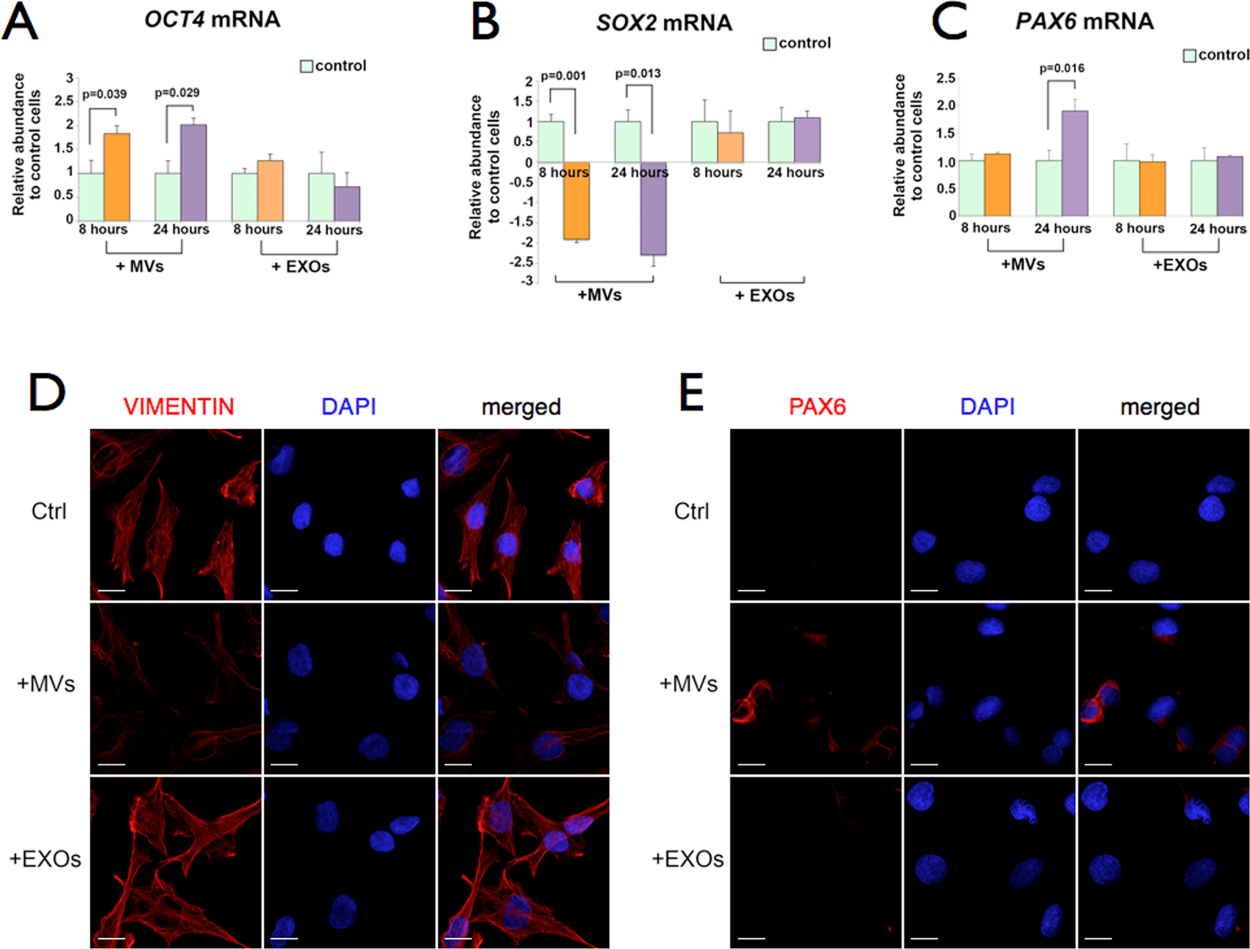
Müller cells’ *OCT4*, *SOX2* and *PAX6* mRNA levels after a single MVs or EXOs treatment. Bar plots show the fold-change in mRNA levels for *OCT4* (A), *SOX2* (B) and the early retinal *PAX6* (C) in Müller cells exposed for 8 and 24 hours to either MVs or EXOs, compared to those in control Müller cells that received culture medium changes only (light green bars). *GAPDH* mRNA was used as a loading control for RT-qPCR. Data are mean ± SEM from 3 independent experiments. Student’s t-test was used to calculate p-values. (D and E) Immunocytochemistry of Müller cells after 13 repeated exposures to MVs or EXOs. Confocal microscopy images of control and MV- or EXO-treated Müller cells immunolabeled with antibodies against VIMENTIN (D, red) or PAX6 (E, red). Cell nuclei were labeled with DAPI. Magnification: 40X, scale bar: 20 μm for all panels.

Cultured Müller cells were subjected to a single exposure of MVs or EXOs. Compared to control Müller cells, MVs increased *OCT4* mRNA level by approximately 1.8-fold as early as 8 hours after exposure, reaching 2-fold by 24 hours (Fig 7A). In contrast, *SOX2* mRNA level was reduced 1.9-fold after 8 hours and reached a lower 2.3-fold decrease at 24 hours (Fig 7B). The expression level of the *PAX6* mRNA did not change in the first 8 hours but it increased by 1.9-fold after 24 hours of treatment (Fig 7C). Exposure to EXOs did not have any significant effect on Müller cells *OCT4*, *SOX2* and *PAX6* mRNA levels at 8 or 24 hours (Fig 7A, 7B and 7C). Similarly to what we observed with repeated hESEV treatments, when Müller cells were cultured adding every other day, for 13 times, freshly isolated MVs or EXOs under the same experimental conditions, only those cells exposed to MVs showed by immunocytochemistry decreased expression of VIMENTIN (Fig 7 D) and an increased expression of PAX6 (Fig 7E). Consecutive exposures to EXOs had no effect on VIMENTIN or PAX6 expression in human Müller cells (Fig 7D and 7E).

## Discussion

We had previously demonstrated that mouse ESMVs transfer mRNA, microRNA, and proteins from the cells from which they are released to different recipient cells (i.e, fibroblasts [2] and glial retinal progenitors Müller cells [11]), altering their gene expression, epigenetic state, and ultimately, their cell fate. We now show that human ESC-derived MVs also have a profound effect on Müller cells exposed to them. To obtain these results, we first performed a detailed characterization of hESEVs and the products of their fractionation, MVs and EXOs.

Although we could maintain H9 hESCs cultures undifferentiated through passage 50, as shown by the stable expression of *OCT4*, *NANOG* and *SOX2* mRNAs, the total RNA content of the released hESEVs decreased by passage 46 and, at passage 50, it was about a fourth of that at passages 38–42. These results were consistently reproduced and may indicate either a reduction in the number of hESEVs released from hESCs at late passages or the inability of these cells to keep packing nanovesicles with the same amount of total RNA. Regardless of the mechanism, the hESEV reduced total RNA content could be an early sign of a progressive decrease, over time, in the quality of H9 stem cells. We thus decided to use for our experiments conditioned medium from H9 hESCs up to passage 44.

During development, pluripotent stem cell fate is mainly determined by OCT4 and SOX2, transcription factors that act as molecular switches to activate or repress expression of specific genes. While OCT4 has been shown to be a master regulator of pluripotency that controls lineage commitment [28], the main role of SOX2 in embryonic stem cells is to keep an appropriate level of OCT4 expression [29, 30]. We assessed the ratios in which *OCT4* and *SOX2* mRNAs are in hESCs and hESEVs and found that they are similar. Thus, we hypothesize that *OCT4* and *SOX2* mRNAs from hESCs are packed into hESEVs with no selective enrichment.

hESEVs are heterogeneous in size and contain MVs and EXOs. MVs are between 10 to 40 times larger than EXOs and have significantly longer RNAs than those present in EXOs, which seem to be enriched in miRNAs. We also tried to differentiate MVs from EXOs by Western blot. As MVs are generated through plasma membrane shedding while EXOs originate from multi-vesicular bodies, we hypothesized that some membrane proteins from hESCs would likely be specifically enriched in MVs compared to EXOs. We thus screened several membrane proteins expressed on the surface of H9 ESCs (data not shown) and found that CD90 is only present on MVs, whereas CD9 and CD63, two known EXO markers [31], are exclusively seen on EXOs. Therefore, we adopted CD90 as a marker for H9 hESC-released MVs. Consistent with the literature, we found that CD81 is present in both MVs and EXOs fractions [20, 31].

Microarray analysis indicated that MVs and EXOs have a large number of differentially expressed mRNAs; the majority of them are at much higher levels in MVs than in EXOs. *OCT4*, *SOX2* and *NANOG*, as well as *SLC7A3* and *THY-1* are examples of these mRNAs. On the other hand, *KLF4*, *HOXA3*, *CECR7* and *PI4K2A* mRNAs are expressed at higher level in EXOs than in MVs. The homeobox gene *HOXA3* codes for a DNA-binding transcription factor particularly involved in the process of differentiation from ES cells to embryonic bodies [32], while most of the other genes are important for the normal functioning of the eye and defects in them have been shown to cause disease. For example, the protein product of *SLC7A3* is a sodium-independent cationic amino acid transporter associated with congenital aphakia, a rare eye disease characterized by the complete lack of the lens and iris [33]. *KLF4* encodes a transcription factor that limits retinal ganglion cell axon bundle thickness [34]. *CECR7* is a ncRNA gene associated with Cat Eye Syndrome, a rare condition caused by trisomy or partial tetrasomy in Chromosome 22. *PI4K2A* (phosphatidylinositol 4-kinase IIα) encodes an enzyme involved in the synthesis of PIP_2_, the phospholipid that sustains a large K^+^ conductance providing a major pathway for K^+^ efflux in the retinal pigment epithelium and Müller cells [35].

Müller cells are the principal glial cells in the retina maintaining homeostasis [36]. Spanning across the full thickness of the retina, Müller cells form a scaffold that supports all retinal neurons; in addition, they form the inner and outer limiting membranes as well as intercellular junctions, and allow the establishment of the retina laminar pattern and polarity [37]. It has been shown that these cells do not irreversibly exit the progenitor state, since they re-enter the cell cycle in response to retinal injury and can give rise to new neurons [38, 39]. Thus, Müller cells are dormant progenitor cells and, as such, are important targets for studies of retinal regeneration.

We before demonstrated that exposure of cultured human Müller cells to mESEVs induces morphological and transcriptome changes in these cells and may reactivate the developmental program involved in retinogenesis [11]. In the current study we show that H9 hESC-derived hESEVs have similar effects, the main differences with mESEVs being the amount of mRNAs transferred and the time needed to cause the corresponding changes. For example, after 24 hours of treatment, the *OCT4* mRNA transferred from hESEVs has increased by 2.5 times the *OCT4* mRNA level in non-exposed cells; in contrast, *Oct4* mRNA from mESEVs reaches maximum transfer into Müller cells much faster, at 8 hours of exposure, with peak levels 27-fold higher than in non-exposed cells [11]. Moreover, the transferred mouse *Oct4* mRNA induces an increase in the endogenous human *OCT4* mRNA level of Müller cells similar to that caused by the transferred human *OCT4* mRNA from hESEVs. If translated, this 2.5–fold increase in *OCT4* mRNA may be enough to induce differentiation of Müller cells to other retinal neurons. Furthermore, *SOX2* mRNA levels decrease from those in control, non-exposed cells by 2.5-fold after 24 hours of Müller cells’ treatment, probably allowing for their de-differentiation and, at the same time, helping to control in the cells the level of *OCT4* mRNA expression. These conditions may be optimal for Müller cells re-entry into the cell cycle, which would keep open, if needed, the possibility of proliferation and reactivation of the cells’ retinogenic program.

hESEV-induced changes in Müller cells’ *OCT4* and *SOX2* mRNAs were corroborated by changes in the corresponding proteins. Comparable results were obtained with cultured Müller cells exposed for 24 hours to MVs: *OCT4* mRNA levels increase by 2.0-fold and those of *SOX2* mRNA decrease by 2.3-fold. Conversely, we found that exposure of Müller cells to EXOs does not change the *OCT4* and *SOX2* mRNA levels in the cells. It is noteworthy to point out that despite the different effects MVs and EXOs have on Müller cells, our uptake experiments showed that Müller cells similarly internalize MVs and EXOs. Thus, our results suggest that mRNA transfer into Müller cells by MVs and EXOs primarily depends on their payload rather than in a differential access to the target cell. They also indicate that the effects observed with hESEV treatments of Müller cells are mostly caused by the MVs present in non-fractionated hESEVs.

The increase in *OCT4* and decrease in *SOX2* mRNAs occurring as a result of Müller cell hESEV exposure may enhance the transcription/translation of early retinal genes. Interestingly, endogenous *PAX6* and *RAX* mRNA levels of human Müller cells are increased over those in non-treated cells after 8 hours treatment with mESEVs [11] and after 24 hours exposure to hESEVs. These results possibly indicate the presence of different control mechanisms in mouse and human ESEVs that allow for accumulation or use and degradation of the corresponding mRNAs. In fact, we show by immunocytochemistry that after 13 iterative exposures to freshly obtained hESEVs, while the Müller cell marker VIMENTIN decreases its expression, confirming that the cells are de-differentiating, expression of the early retinal protein PAX6 increases, suggesting the possible trans-differentiation of Müller cells into retinal neuronal precursors. Similar results are observed by exposing the cultured Müller cells to MVs, but not to EXOs, again demonstrating that MVs in hESEVs are responsible for all the effects that we have described. As with mESEVs and in spite of *NANOG’s* presence in hESEVs, we did not find its mRNA transferred into Müller cells after their exposure to hESEVs, suggesting that either the transfer process is selective for mRNAs and *NANOG* is excluded or that *NANOG* mRNA is rapidly degraded once it is in the Müller cells.

## Conclusion

Our results have shown that human embryonic stem cell-derived MVs are responsible for the glial to neuronal trans-differentiation of cultured Müller cells exposed to them. Thus, we ask: What happens in the living retina? Do MVs induce *in vivo* de-differentiation of quiescent glial Müller cell progenitors to a stem cell phenotype, cell cycle re-entry, changes in Müller cell’s microenvironment towards a more permissive state for tissue regeneration and a retinogenic program leading to regeneration? I*n vivo* studies may answer these questions and definitively demonstrate that MVs are promising therapeutic agents for the rescue of degenerating or damaged retinas.

## Supporting information

S1 Fig Expression of pluripotency mRNAs and corresponding proteins in H9 hESCs from passages P38 to P50, and dependence of the total RNA level of released hESEVs on hESCs’ passage number. (A) qPCR measurements show similar levels of pluripotency mRNAs *OCT4*, *SOX2* and *NANOG* in H9 cells at different passages. (B and C) Immunocytochemical detection of pluripotency-maintaining proteins TRA1-81, SOX2, OCT4 and NANOG in H9 hESCs at passages 41 (B) and 50 (C). Upper row: double immunostaining with TRA1-81 (green) and SOX2 (red) antibodies; middle and bottom rows: immunostaining with OCT4 (red) or NANOG (red) antibodies, respectively. Cell nuclei were counterstained with DAPI (blue). Magnification: 10X, scale bar: 200 μm for all panels. (D) Total RNA from hESEVs derived from different H9 hESC passages. All measurements were repeated 6 times. Error bars represent the SEM; p = 0.0008 was determined by statistical analyses using a repeated measures ANOVA model for the overall mRNA level difference across all groups. Significant differences between pairs of samples were determined using the unpaired Student’s t-test and are indicated by the p values shown on the horizontal lines marking the two compared groups.
(TIFF)

S2 Fig Representation of specific cell functions and diseases associated with the 3,724 genes differentially expressed in MVs and EXOs at p<0.05 and fold-change ≥ 2. Significant association versus random change association of these genes with specific cell functions and diseases was tested in the total curated database of gene interactions of over 23,900 human, rat and mouse genes by the Right-tailed Fisher’ exact test (Ingenuity Systems).
(TIFF)

S3 Fig Representation of canonical cell signaling pathways associated with the 3,724 genes differentially expressed in MVs and EXOs at p<0.05 and fold-change ≥ 2. These genes also were tested for significant association versus random change association with canonical cell signaling pathways like EIF2 signaling (regulates both global and specific mRNA translation), mTOR signaling (controls key cellular processes such as cell survival, growth and proliferation), VEGF signaling (regulates vascular development in the embryo) and HIPPO signaling (involved in restraining cell proliferation and promoting apoptosis), in a total curated database of gene interactions of over 23,900 human, rat and mouse genes by Right-tailed Fisher’s exact test (Ingenuity Systems). The orange line indicates the threshold for a significant association.
(TIFF)
