# Supplementary figures and images for: Human embryonic stem cells extracellular vesicles and their effects on immortalized human retinal Müller cells
Source: PLoS One. 2018 Mar 14;13(3):e0194004. doi: 10.1371/journal.pone.0194004 (PMC5851617; doi:10.1371/journal.pone.0194004)

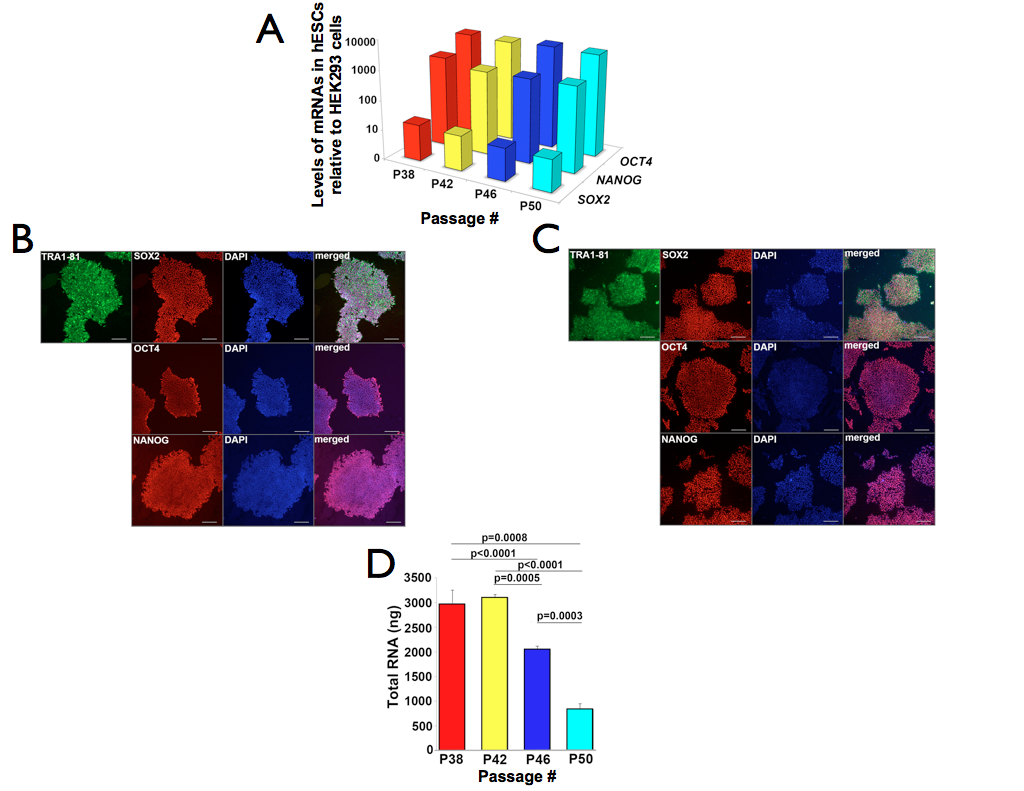

Supplement: S1 Fig — (A) qPCR measurements show similar levels of pluripotency mRNAs OCT4, SOX2 and NANOG in H9 cells at different passages. (B and C) Immunocytochemical detection of pluripotency-maintaining proteins TRA1-81, SOX2, OCT4 and NANOG in H9 hESCs at passages 41 (B) and 50 (C). Upper row: double immunostaining with TRA1-81 (green) and SOX2 (red) antibodies; middle and bottom rows: immunostaining with OCT4 (red) or NANOG (red) antibodies, respectively. Cell nuclei were counterstained with DAPI (blue). Magnification: 10X, scale bar: 200 μm for all panels. (D) Total RNA from hESEVs derived from different H9 hESC passages. All measurements were repeated 6 times. Error bars represent the SEM; p = 0.0008 was determined by statistical analyses using a repeated measures ANOVA model for the overall mRNA level difference across all groups. Significant differences between pairs of samples were determined using the unpaired Student’s t-test and are indicated by the p values shown on the horizontal lines marking the two compared groups. (TIFF) [file pone.0194004.s001.tiff]

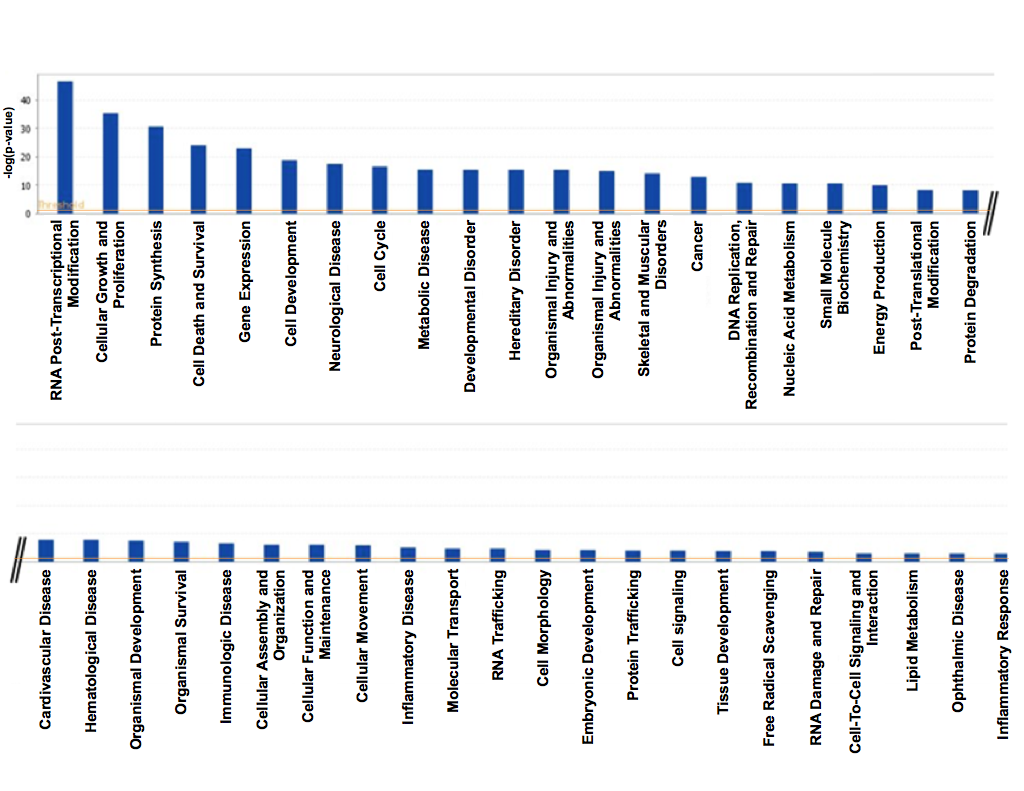

Supplement: S2 Fig — Significant association versus random change association of these genes with specific cell functions and diseases was tested in the total curated database of gene interactions of over 23,900 human, rat and mouse genes by the Right-tailed Fisher’ exact test (Ingenuity Systems). (TIFF) [file pone.0194004.s002.tiff]

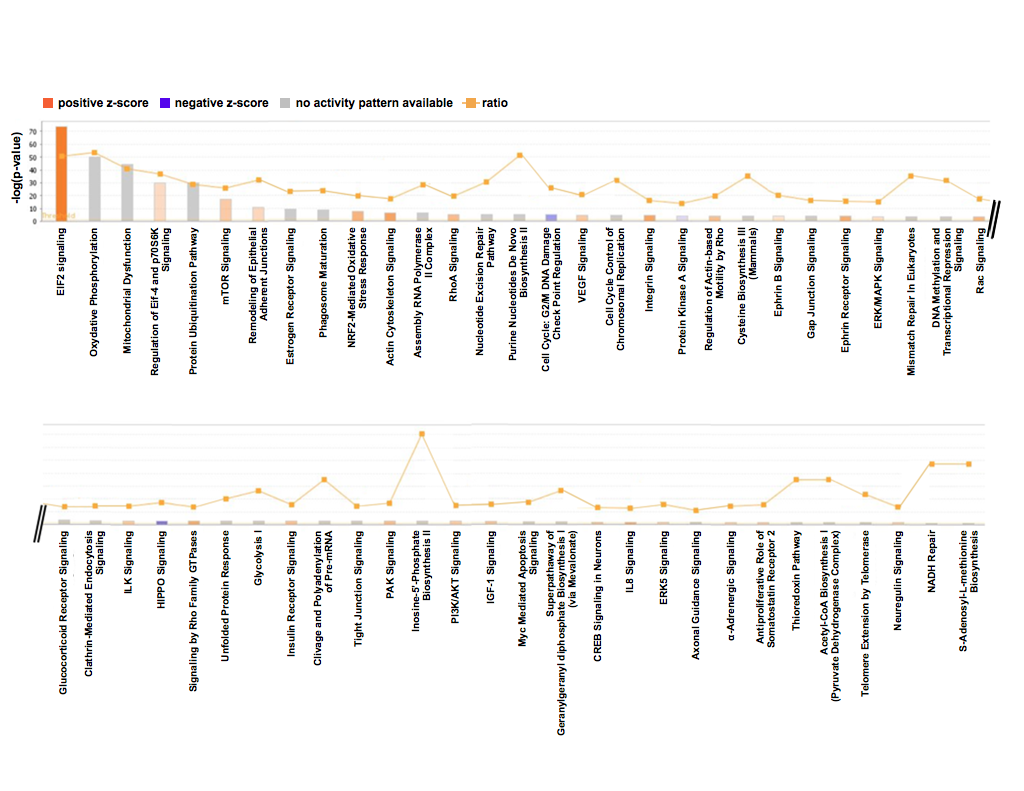

Supplement: S3 Fig — These genes also were tested for significant association versus random change association with canonical cell signaling pathways like EIF2 signaling (regulates both global and specific mRNA translation), mTOR signaling (controls key cellular processes such as cell survival, growth and proliferation), VEGF signaling (regulates vascular development in the embryo) and HIPPO signaling (involved in restraining cell proliferation and promoting apoptosis), in a total curated database of gene interactions of over 23,900 human, rat and mouse genes by Right-tailed Fisher’s exact test (Ingenuity Systems). The orange line indicates the threshold for a significant association. (TIFF) [file pone.0194004.s003.tiff]
